# Effectiveness of Photobiomodulation Versus Local Drug Delivery as an Adjunct to Non-surgical Periodontal Therapy in the Management of Periodontal Pockets: A Case Report

**DOI:** 10.7759/cureus.68719

**Published:** 2024-09-05

**Authors:** Anju Rana, Pooja Palwankar, Lipika Gopal

**Affiliations:** 1 Periodontology, Manav Rachna Dental College, School of Dental Sciences, Manav Rachna International Institute of Research and Studies (MRIIRS), Faridabad, IND

**Keywords:** ldd, non-surgical periodontal therapy, photobiomodulation, pocket therapy, tetracycline fibers

## Abstract

Periodontitis, a bacterial infection leading to the destruction of tooth-supporting tissues, is primarily driven by elevated levels of subgingival microorganisms. Effective periodontal therapy aims to eliminate or reduce these pathogens to halt disease progression, prevent recurrence, and regenerate lost tissues. This case report evaluates the effectiveness of non-surgical therapies, specifically tetracycline fibers and photobiomodulation therapy (PBMT), as adjuncts to scaling and root planing (SRP) in the treatment of periodontal disease using a split-mouth design. Mechanical SRP alone may not fully remove pathogens from deep periodontal pockets, potentially leading to disease recurrence. To address this, various other techniques have been explored, including the use of tetracycline fibers and PBMT. Local delivery methods aim to minimize systemic side effects while maintaining high antimicrobial concentrations in the gingival crevicular fluid, whereas PBMT, known for its anti-inflammatory and regenerative properties, involves exposing tissues to low-intensity light with wavelengths from red to near-infrared. This therapy activates cellular functions and mitochondrial processes, resulting in increased adenosine triphosphate (ATP) production, nitric oxide levels, and overall tissue healing. Tetracycline fibers have been highlighted for their effectiveness in periodontal therapy due to their localized antimicrobial action. The integration of tetracycline fibers and PBMT with SRP presents a promising non-surgical approach to enhance the management of periodontal disease. The case report aims to provide insights into the efficacy of these adjunctive therapies in improving clinical outcomes for patients with periodontitis.

## Introduction

Periodontitis is a bacterial infection that leads to the destruction of the tissues supporting the teeth. There is substantial evidence indicating that bacteria are central to the development of periodontal disease, with increased levels of subgingival microorganisms linked to the progression of destructive periodontal activity [1]. A key aim of periodontal therapy is to eliminate or reduce these microbial pathogens in subgingival plaque. Identifying the roles of specific bacteria in chronic periodontitis has refined therapeutic strategies for managing the condition. Thus, effectively removing or inhibiting subgingival plaque is essential for maintaining oral health. The primary goals of periodontal therapy are to halt disease progression, prevent recurrence, and regenerate lost tissues.

Mechanical debridement alone may not fully eradicate pathogens from periodontal pockets due to their deep location within gingival tissue or inaccessible areas, potentially leading to disease recurrence [1]. Therefore, combining mechanical scaling and root planing (SRP) with selective antimicrobial treatments or low-level laser therapy is often considered an effective non-surgical approach for targeting active disease sites.

Systemic antibiotics circulate throughout the body but are limited by potential adverse reactions, including toxicity, bacterial resistance, drug interactions, and patient compliance issues [2]. To address these challenges, various local delivery methods have been developed to administer antimicrobial agents directly into periodontal pockets. Local delivery minimizes systemic side effects and maintains high concentrations of antimicrobial agents in the gingival crevicular fluid over extended periods, often achieving levels higher than those possible with systemic administration [1,3]. Tetracycline has received significant attention among antimicrobials used in periodontal therapy.

Another non-surgical technique, photobiomodulation therapy (PBMT), previously known as “low-level laser therapy,” involves exposing tissues and cells to non-ionizing, low-intensity light with wavelengths ranging from red (600-700 nm) to near-infrared (700-1400 nm). This light, absorbed by endogenous chromophores, induces various biological effects. PBMT was first introduced by Endre Mester in 1968, and NASA subsequently developed it to promote muscle cell healing and regeneration in astronauts [4]. Recent decades have seen a surge in research into PBMT, underscoring its growing therapeutic potential [5].

PBMT’s primary mechanism involves the mitochondria, which contain photo-acceptors responsive to the light wavelengths used. PBMT activates non-mitochondrial cellular functions (such as light/heat-gated ion channels) and restores mitochondrial function (via interfacial water and/or cytochrome C oxidase activation), leading to increased adenosine triphosphate (ATP) production and nitric oxide levels [4]. These effects result in long-term benefits, including the expression of various stimulatory and protective genes. Studies have demonstrated PBM’s anti-inflammatory and analgesic properties, as well as its benefits in enhancing blood circulation, angiogenesis, and tissue healing/regeneration [4].

Currently, there are no case reports or studies comparing PBMT with tetracycline fibers as adjuncts to SRP. To address this gap, we present a case report utilizing a split-mouth technique with consistent conditions and probing depths around the same tooth, to assess which non-surgical adjunct - PBMT or tetracycline fibers - is superior for the treatment of periodontal disease.

## Case presentation

A 26-year-old female patient presented to the outpatient department of periodontology with a complaint of bleeding gum persisting for the past two months. The patient was otherwise healthy, with no underlying systemic conditions affecting her periodontal or dental health. She was diagnosed with localized stage II grade A periodontitis according to the 2017 World Workshop on the Classification of Periodontal and Peri-Implant Diseases and Conditions [6].

**Figure 1:**
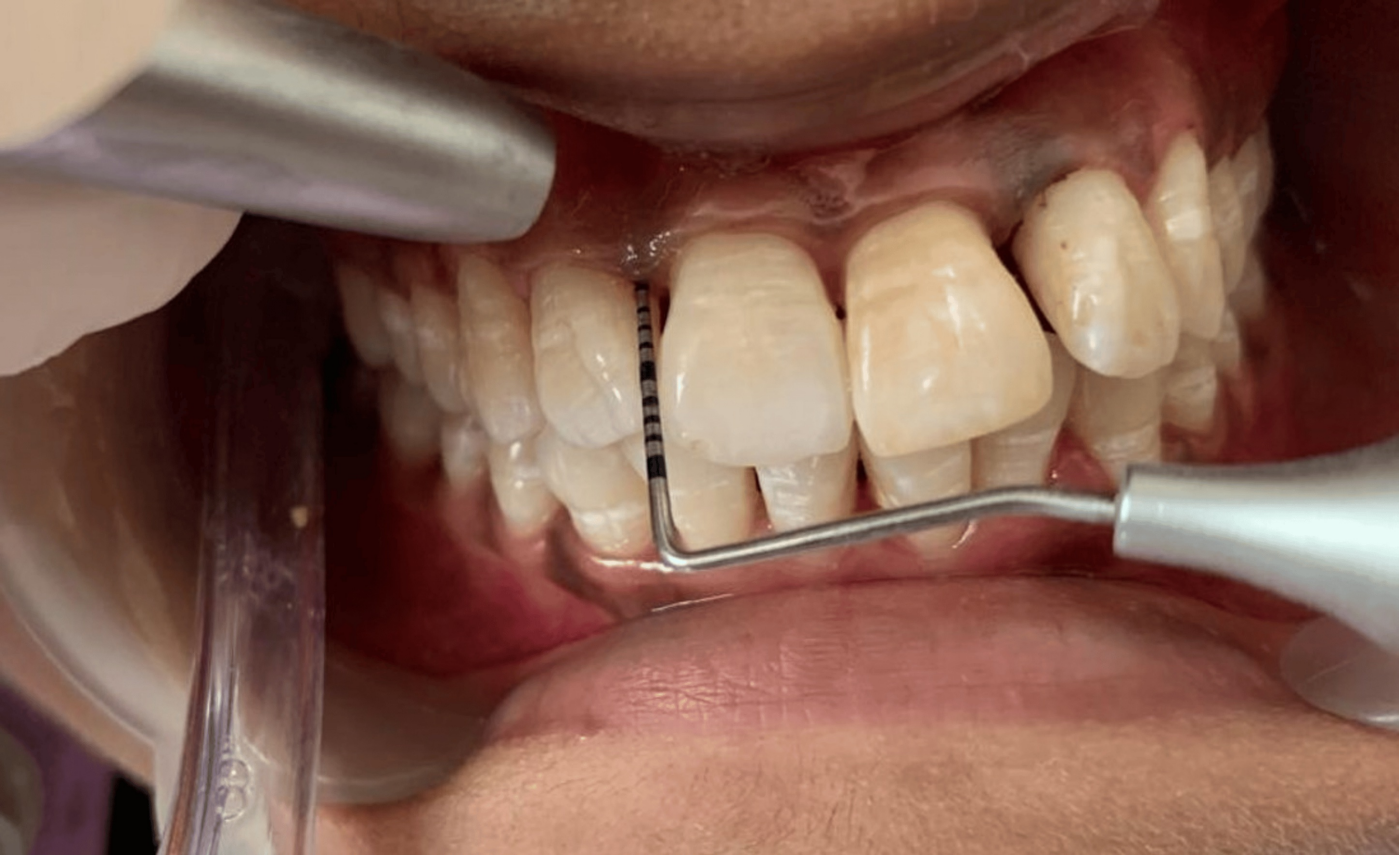
Pre-operative CAL in relation to 12 (mesiobuccal aspect). CAL: clinical attachment loss

Initial treatment included complete ultrasonic scaling of the maxillary and mandibular arch. Generalized periodontal probing depth was evaluated and measured around teeth 12, 16, 22, and 26 on the mesio-buccal, mid-buccal, disto-buccal, and palatal surfaces, revealing bilateral similar mesio-buccal pockets at these sites (Figure 1).

**Figure 2:**
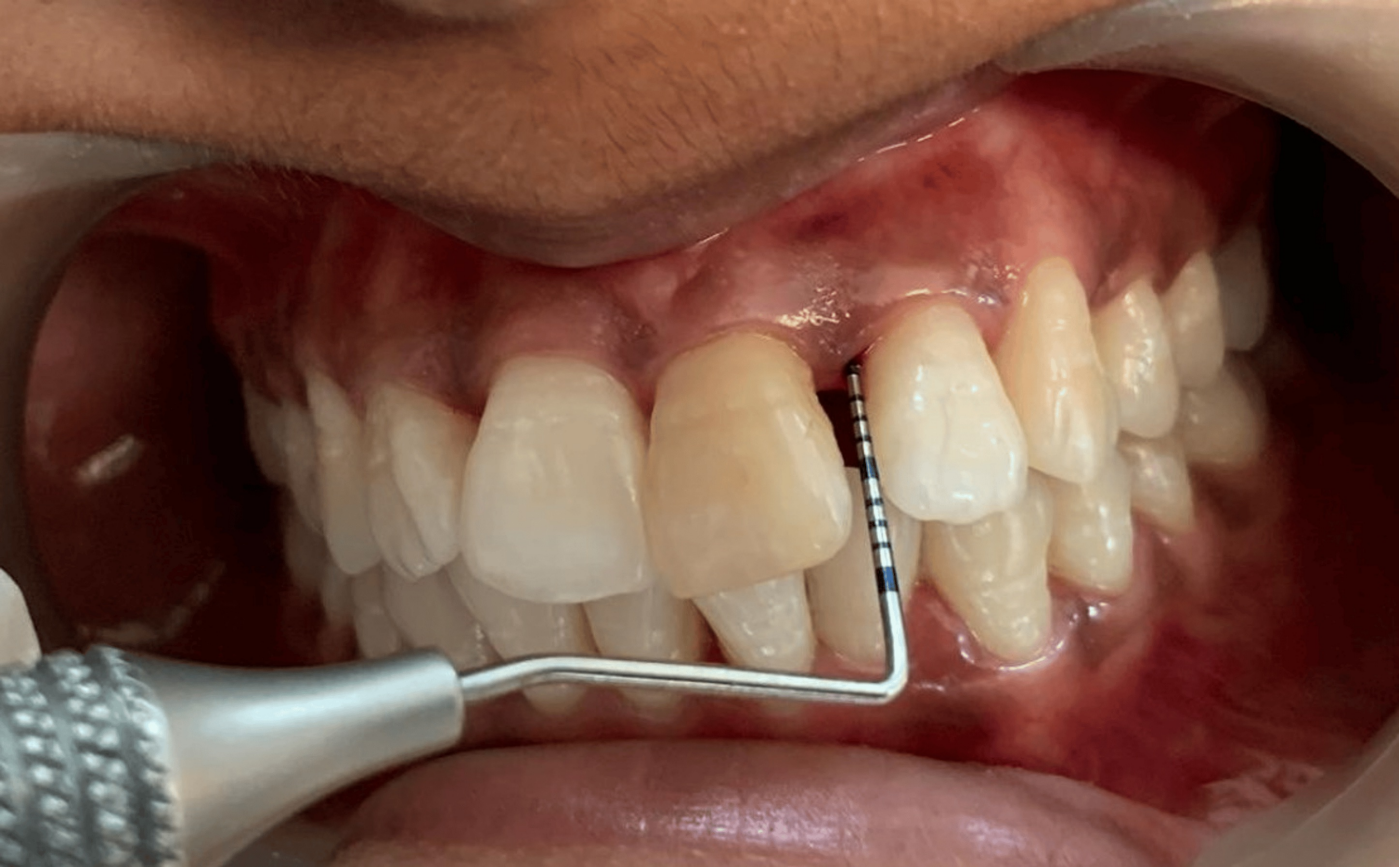
Pre-operative CAL in relation to 22 (mesiobuccal aspect). CAL: clinical attachment loss

**Figure 3:**
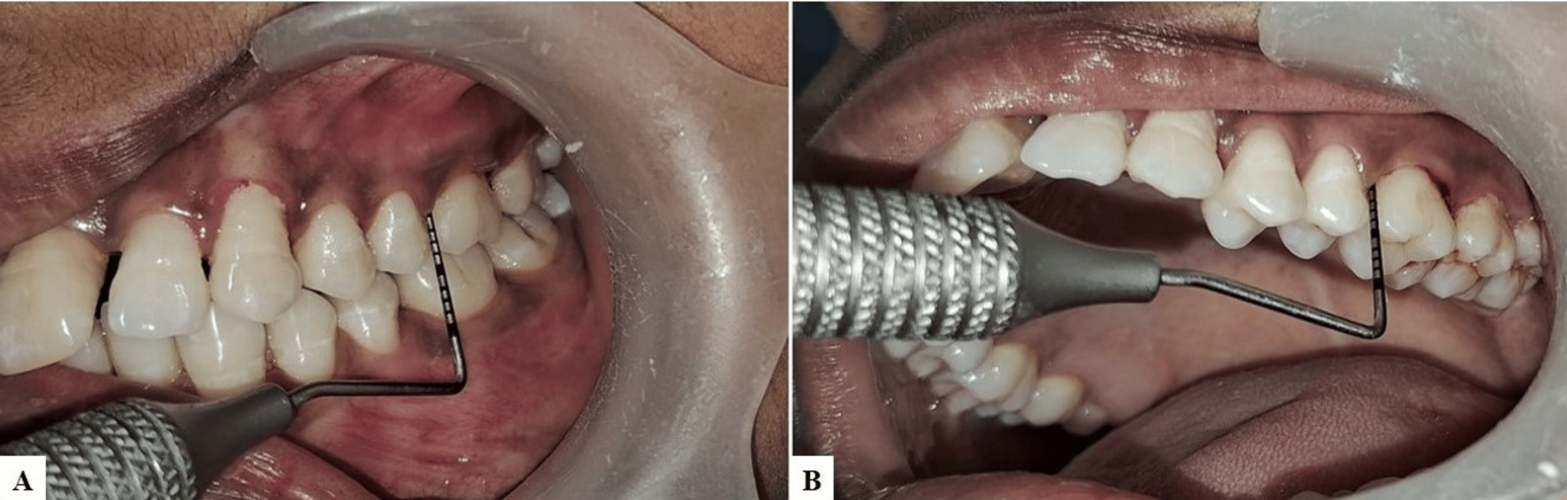
(A) Pre-operative mesio-buccal CAL in relation to 26 using calibrated UNC-15 periodontal probe (buccal view). (B) Pre-operative mesiobuccal CAL in relation to 26 using calibrated UNC-15 periodontal probe (occlusal view). CAL: clinical attachment loss

A split-mouth design was employed. The right maxillary lateral incisor and first molar and left maxillary lateral incisor and first molar with periodontal pockets measuring ≥5 mm were selected for different treatment modalities (Figures 2, 3).

These sites were randomly assigned as follows: Site A received SRP followed by placement of tetracycline fibers, while Site B was treated with SRP followed by PBMT. Clinical parameters including gingival index, pocket probing depth, and clinical attachment level (CAL) were recorded at baseline, 30 days, 90 days, and 180 days.

Prior to any intervention, a non-surgical treatment plan involving PBMT and local drug delivery was discussed and explained to the patient. The patient was informed about the details of the treatment, potential side effects, and benefits. Preferring non-surgical options, the patient provided written informed consent to proceed. Preoperative intraoral photographs and periapical radiographs were taken for documentation.

The patient was given instructions on oral hygiene, including proper brushing techniques and the use of interdental brushes and dental floss. SRP was performed using an ultrasonic piezoelectric scaler (Woodpecker DTE D5 with Optic Handpiece). Following the initial scaling, root planing was completed using universal and Gracey curettes after one week of subgingival scaling, and the sulcus was irrigated with 0.2% chlorhexidine mouthwash for approximately 10 seconds via an endodontic syringe needle tip. At the follow-up visit after root planing, there were no signs of gingival inflammation, and the periodontium appeared healthy.

**Figure 4:**
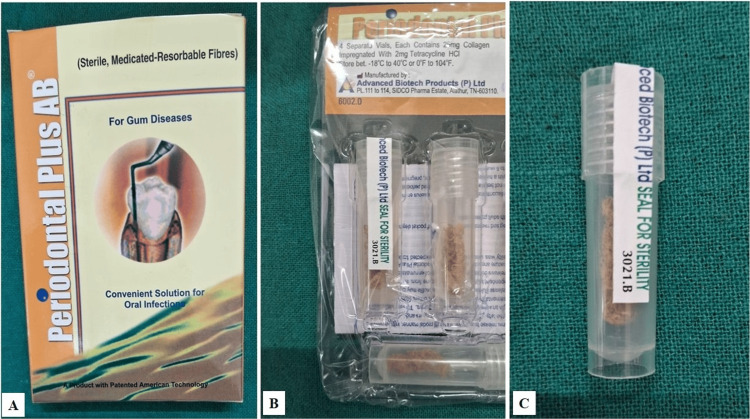
(A) Tetracycline fibers (Periodontal Plus AB) used as local drug delivery agent for non-surgical periodontal therapy. (B) Sterile sealed three vials of tetracycline fibres. (C) Single vial of commercially available tetracycline fibers (Periodontal Plus AB) used for non-surgical treatment.

The non-surgical treatment adhered to the European Federation of Periodontology (EFP) guidelines. Tetracycline fibers (Figure 4) were applied to the left side (Site A), and PBMT was administered to the right side (Site B).

**Figure 5:**
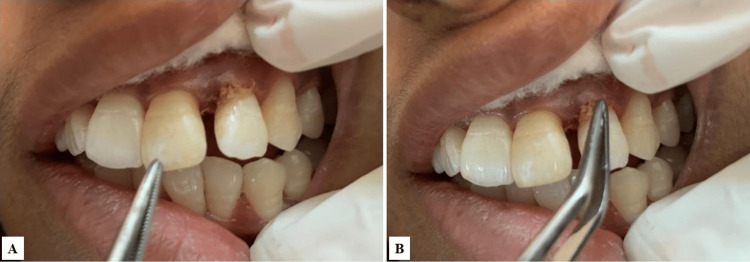
(A) Tetracycline fibers (Periodontal Plus AB) in the mesiobuccal pocket of the left lateral incisor. (B) Partial placement and insertion of tetracycline fibers using a tweezer and UNC-15 periodontal probe in the mesiobuccal pocket of the left lateral incisor.

Periodontal Plus AB (sterile medicated resorbable fibers) were placed in the mesiobuccal pockets of the maxillary left lateral incisor and first molar using a tweezer (Figure 5).

**Figure 6:**
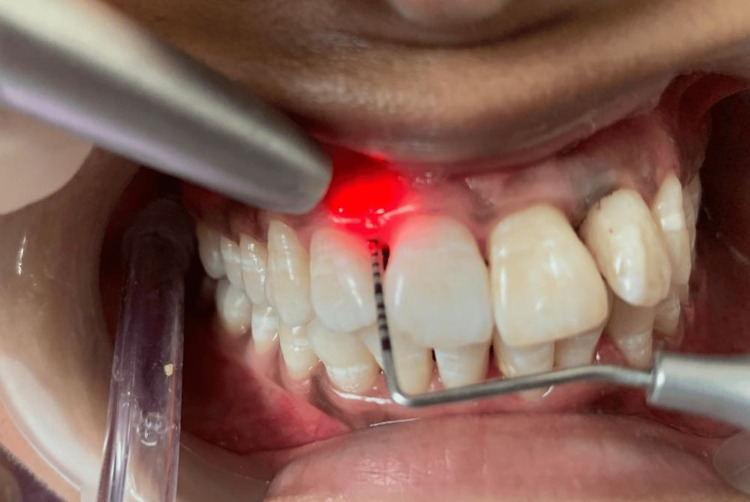
Photobiomodulation using a diode laser in relation to the mesiobuccal pocket of the right lateral incisor.

**Figure 7:**
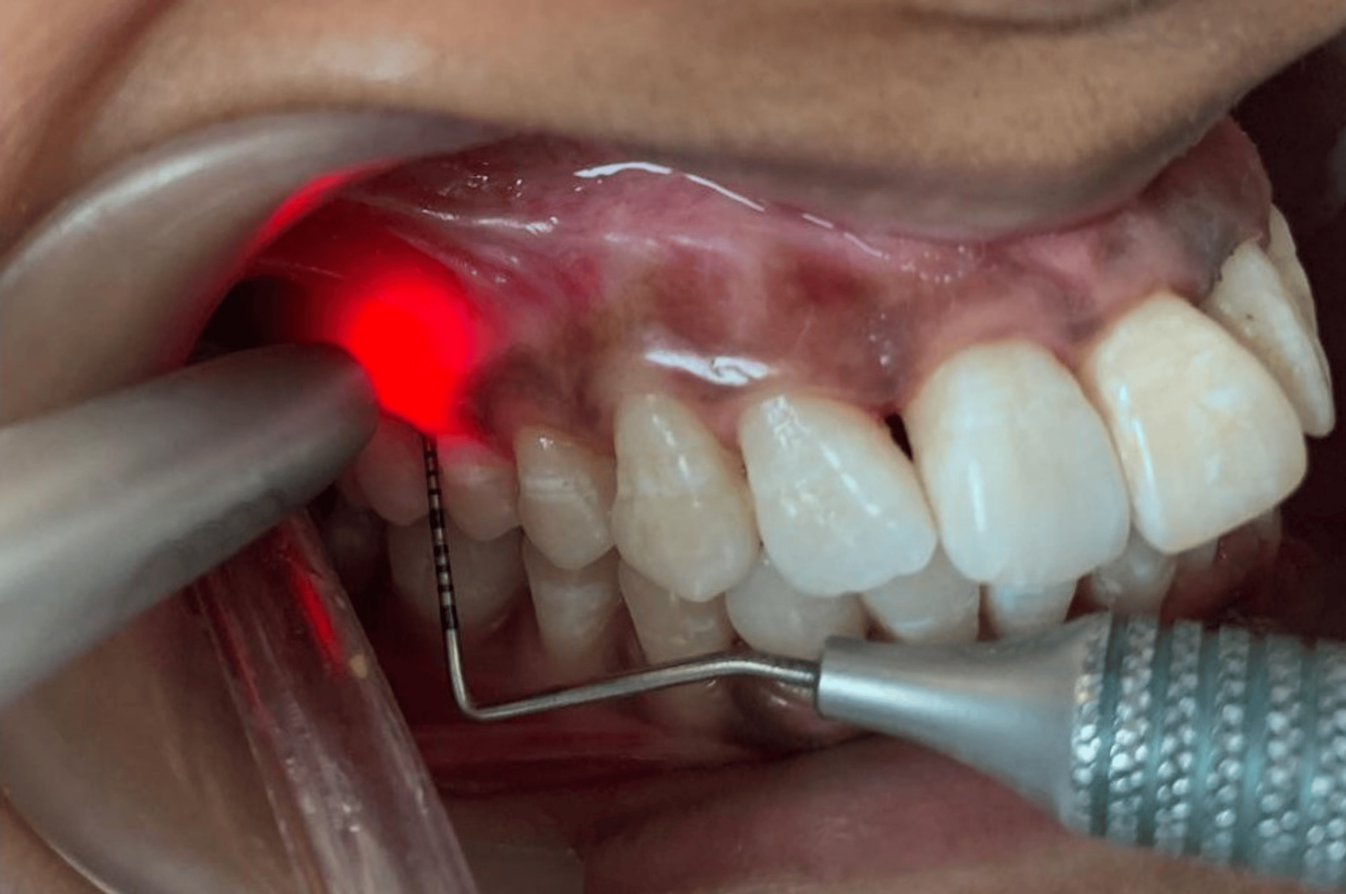
Photobiomodulation therapy with a diode laser with respect to the mesiobuccal pocket of the right first molar.

It was ensured that the complete pocket was filled with the fibers. On the other side, the PBMT was conducted with a 940 nm wavelength diode laser (Biolase epic) following specific protocols. During the first session, PBM was applied at four points: between the central and lateral incisors on the right side (Figure 6) and between the second premolar and first molar in the first quadrant (Figure 7) - targeting the coronal third, apical third, and mesial and distal papillae, respectively.

**Figure 8:**
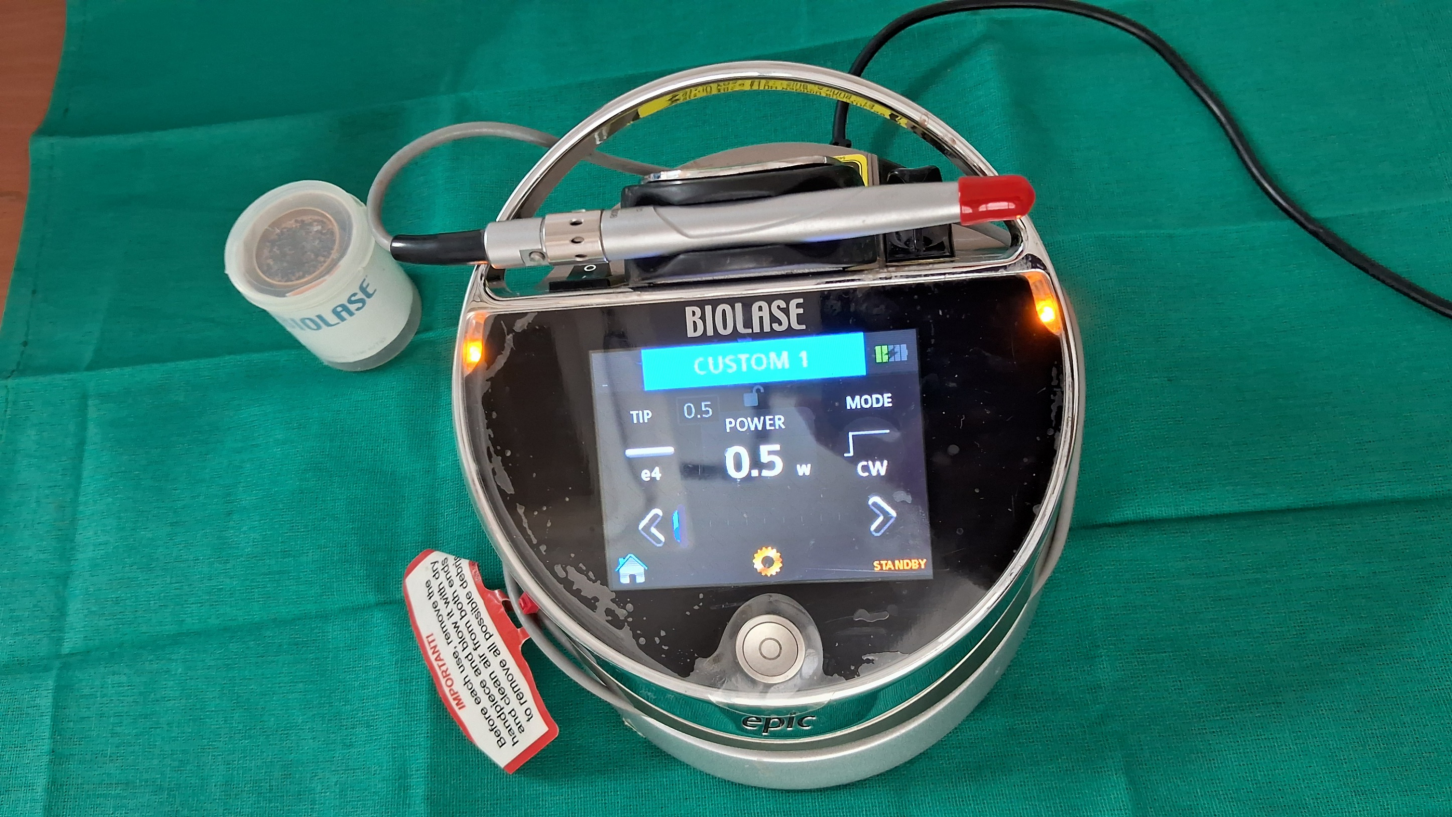
Biolase epic laser specifications used for the photobiomodulation therapy.

PBMT was reapplied at the same four points three times in an interval of 10 minutes each. Parameters included continuous mode, non-contact mode, 940 nm wavelength, spot size of 8 mm, irradiation time of 50-60 seconds per point, power output of 0.5 W continuous wave (Figure 8).

**Figure 9:**
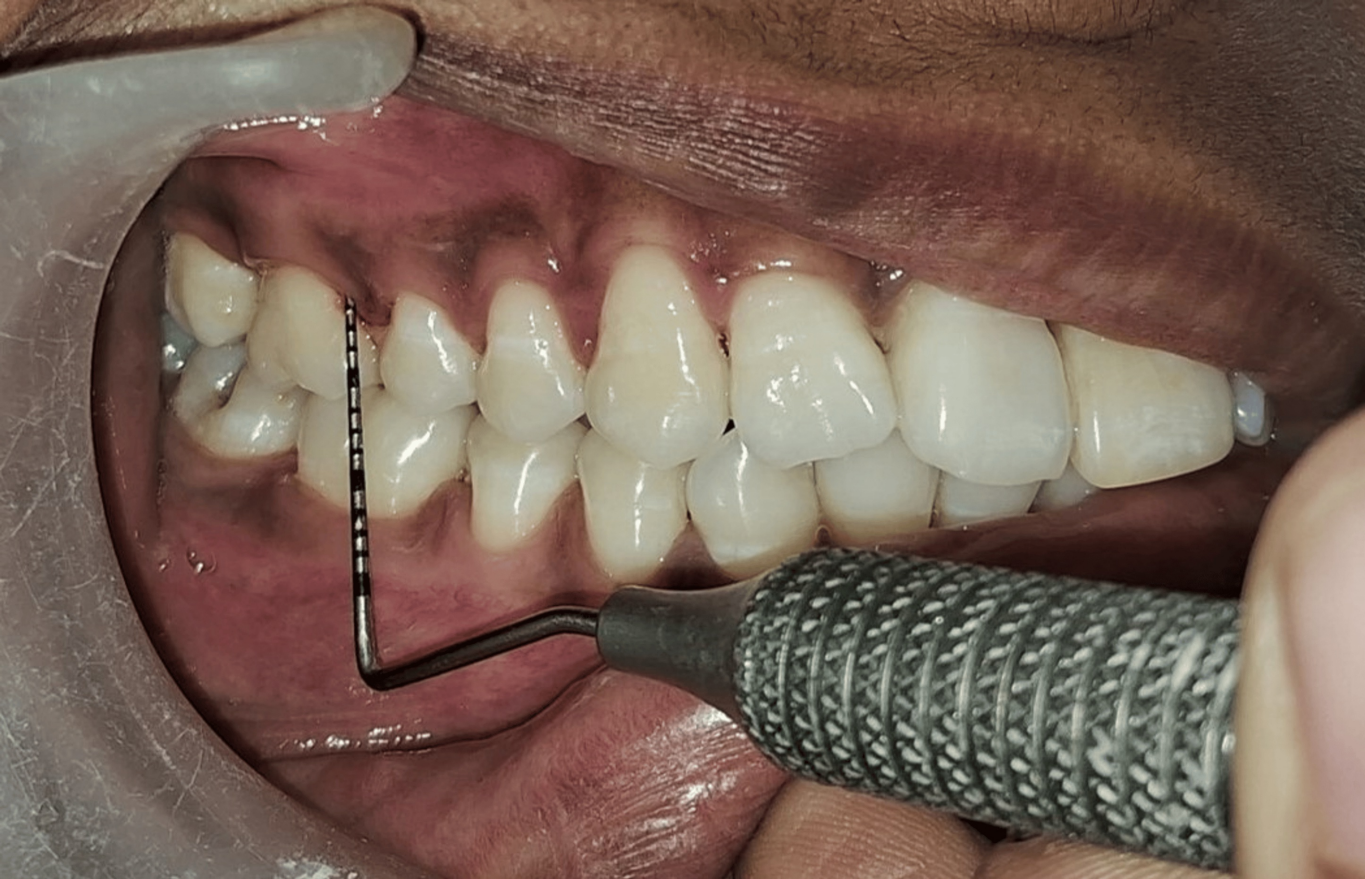
Reduced post-operative probing depth after six months with respect to the right maxillary first molar.

Post-operative instructions included avoiding brushing for 48 hours, refraining from mouthwash use, and abstaining from chewing for two hours. Follow-up appointments were scheduled at one month, three months, and six months post-treatment. Periodontal probing depth was re-evaluated after three months and six months. Three months follow-up showed reduced probing depth to 3 mm wrt right and left lateral incisor and 2 mm wrt right and left first molar. The findings of the probing depth remained the same at six months follow-up (Figure 9).

## Discussion

Tetracycline, known for its broad-spectrum antimicrobial activity, is a valuable adjunct in periodontal therapy. Effective against Gram-positive bacteria, spirochetes, and various anaerobic and facultative bacteria, tetracycline achieves high concentrations in gingival crevicular fluid, enhancing its efficacy in periodontal treatment. Beyond its antimicrobial properties, tetracycline inhibits collagenase, reducing collagen breakdown in periodontal disease, and has been shown to possess anti-inflammatory effects, inhibit bone resorption, and promote fibroblast attachment. These multifaceted benefits make tetracycline a promising addition to non-surgical periodontal therapies [7]. The results of our case report align with the findings of Panwar M et al. and Sadaf N et al., who demonstrated in their research that using tetracycline fibers in addition to scaling and root planing significantly enhances the reduction of inflammation compared to scaling and root planing alone. They found that tetracycline fibers are a simple, effective, non-surgical approach to improving periodontal health, suitable for use even by general dental practitioners [3,8]. Various systems for delivering tetracycline to periodontal pockets exist, including fibers and films. The "Periodontal Plus AB™" system, combining tetracycline HCL with fibrillar collagen, offers a novel approach. For the ease of placement of fibers and retention ability in the pocket, tetracycline fibers were used.

PBMT, initially popularized for aesthetic and sports recovery applications, has gained traction in promoting tissue healing, reducing inflammation, and mitigating the side effects of other treatments. PBMT has shown potential in wound healing, bone defect repair, and periodontitis, with recent research exploring its impact on neurodegenerative and metabolic diseases [5,9].

In this case presentation, combining tetracycline fibers with SRP led to significant clinical improvements in periodontal parameters and a gain in clinical attachment levels. The tetracycline fibers showed efficacy after thorough subgingival debridement, suggesting their antimicrobial and anti-collagenase properties contribute to positive outcomes. Despite the promising results, potential side effects of tetracycline fibers, such as allergic reactions and discomfort, need consideration, although no adverse reactions were reported in this case. Moreover, a limitation of tetracycline use in medically compromised patients paves a new treatment modality as an adjunct to SRP.

PBMT demonstrated positive effects on periodontal healing in preclinical and clinical studies, including enhanced angiogenesis, fibroblast activity, and reduced inflammation [4,10,11]. The variability in PBMT parameters across studies highlights the need for standardized protocols [12]. While promising, further research is necessary to optimize PBMT parameters and establish its definitive role in periodontal therapy. Castro Dos Santos N et al. examined the local effects of PBMT on treating periodontal pockets in patients with periodontitis and type 2 diabetes [9]. They randomly assigned selected periodontal pockets to either receive mechanical debridement alone (control group) or mechanical debridement combined with the PBMT group. At the six-month mark, the PBMT group had a significantly lower frequency of pockets with probing depths of 5-6 mm compared to the control group. In addition, PBMT proved more effective in reducing the moderate periodontal pockets percentage in patients with type 2 diabetes. These findings are consistent with our study, which also demonstrated clinical improvement with PBMT in moderate probing depths [9].

Overall, both tetracycline fibers and PBMT demonstrated potential as adjuncts to SRP for managing periodontitis. The results highlight that non-surgical treatments, rather than surgical ones, may alter patient preferences by avoiding the pain often associated with surgical periodontal procedures, which many people fear and thus frequently avoid. This case emphasizes the need for personalized non-surgical treatment approaches and calls for further research to refine and validate these modalities for long-term clinical application.

## Conclusions

In this case report, both PBMT and tetracycline fibers demonstrated improved outcomes as adjuncts to nonsurgical therapy. Both treatments play a significant role in the non-surgical management of periodontal pockets, yet each has its own limitations and indications. Therefore, the choice of procedure should be guided by specific indications and patient needs, with the clinician making a well-justified decision. The limitations of the present case report include the absence of long-term follow-up up to one year and the reliance on a single case presentation, which limits the ability to generalize the findings. Hence, future research should involve larger sample sizes and long-term follow-up, ideally with randomized trials.
